# The New Caledonian genus *Caledonotrichia* Sykora (Trichoptera, Insecta) reviewed, with descriptions of 6 new species

**DOI:** 10.3897/zookeys.287.4615

**Published:** 2013-04-11

**Authors:** Alice Wells, Kjell Arne Johanson, Nathalie Mary-Sasal

**Affiliations:** 1 Australian Biological Resources Study, PO Box 787, Canberra, ACT 2601 Australia; 2 Entomology Department, Swedish Museum of Natural History, Box 50007, SE-104 05 Stockholm, Sweden; 3 B.P. 271, 98728 Maharepa Moorea, French Polynesia

**Keywords:** Spicipalpia, Hydroptilidae, New Caledonia, endemic, key, generic relationships

## Abstract

The New Caledonian endemic hydroptilid genus *Caledonotrichia* Sykora (Trichoptera) is reviewed and 6 new species are described: *Caledonotrichia bifida*, *Caledonotrichia capensis*, *Caledonotrichia minuta*, *Caledonotrichia ouinnica*, *Caledonotrichia sykorai* and *Caledonotrichia vexilla*. Together with the established species for which revised diagnoses are given, these raise to 11 the number of species known in this genus. The new species, females of 3 species, and several unusual larval cases are examined and described for further insight into relationships of this enigmatic genus. A key to species is provided.

## Introduction

The Trichoptera fauna of freshwaters on the small south-western Pacific island of New Caledonia exhibit high levels of endemism at the species level. In addition, 7 genera are endemic, among which is one hydroptilid genus, *Caledonotrichia* Sykora, 1967. As part of a revision of all New Caledonian Hydroptilidae (Wells & Johanson 2012, and in prep.), this genus is reviewed here. The first 2 species in the genus *Caledonotrichia* Sykora were C. *illiesi* Sykora, 1967and *Caledonotrichia minor* Sykora, 1967, both from a locality near Col d’Amieu in the Province Sud (southern province). Nothing more was published on the genus until J. Marshall in her 1979 review of Hydroptilidae included a brief diagnosis and, not surprisingly given the features identified as diagnostic, was unable to assign the genus to any of the Hydroptilinae tribes that she recognised. In 1989, 2 additional species of *Caledonotrichia* were described by Kelley: *Caledonotrichia charadra* Kelley, 1989, smallish and rather similar to *Caledonotrichia minor*, and a distinctive, much larger-bodied species, *Caledonotrichia extensa* Kelley, 1989. Next, several larvae were described by Wells (1995): a curious early instar larva with greatly modified head setae — cephalic ‘horns’, and mature larvae with dome-shaped cases, both attributed to *Caledonotrichia illiesi*, as well as an unassociated early larva without cephalic horns. Oláh & Johanson (2010) added a fifth species, *Caledonotrichia nyurga* Oláh & Johanson, 2010. Adults of several additionalnew species in *Caledonotrichia* were collected by A. Wells in late 1998, and extensive light- and Malaise-trapping by K.A. Johanson and colleagues from 2001–2006 yielded others. This present study is based mainly on these two collections, although between 1996 and 2000, N. Mary collected extensively in her study on macroinvertebrates of New Caledonian streams (Mary 2002). Mary’s surveys concentrated on aquatic stages and, as few pharate adults were among the hydroptilids collected, most specimens were identifiable only to genus. This was unfortunate since in addition to the fixed dome-shaped cases of the form described by Wells (1995), the samples included further unusual larval cases, identifiable as those of species of *Caledonotrichia*. These are purse-shaped and cylindrical cases constructed of 2 equal valves, with distinct dorsal and ventral sides, some with dorsal vents. One purse-shaped form is made of sections of moss microphylls placed flat in a manner similar to that described by Cairns & Wells (2008) for *Scelotrichia willcairnsi* Cairns & Wells, 2008, a NE Queensland Stactobiini species, that not only makes its case from moss microphylls, but also feeds upon them. These New Caledonian cases differ, however, in having towards each end of one seam, a protruding ‘stalk’, presumably for attachment to the substratum; on the outer side of each stalk is a well-defined opening facing towards the end of the case. A pharate adult female has 34 antennal segments. Among known species only *Caledonotrichia illiesi* and *Caledonotrichia nyurga* have antennae with so many segments, and *Caledonotrichia illiesi* has dome-shaped cases. This may be the case of *Caledonotrichia nyurga*. A second purse-shaped case also made of moss microphylls but this time without dorsal vents, is constructed of small moss microphylls, all bristling out neatly from the surface of the case and giving it a porcupine- or echidna-like appearance. The larvae conform with those of *Caledonotrichia*, but no further identification is possible. The third case type, identified from pharate adults, is that of *Caledonotrichia extensa*. These cases (Fig. 70) comprise two valves, both smoothly rounded, and constructed of silk secretion only. They more or less form a cylinder, but are tapered and flattened towards each end, terminating in a bract-like overhang. Each end has a pair of triangular prominences on the upper seam around openings that face towards each end of the case. Similar dorsal prominences shielding vents occur on the cases of 3 Australian species (see Wells 1997): in *Hellyethira forficata* Wells, 1997, an otherwise ovoid case constructed of sand grains and silk; *Orthotrichia armata* Wells, 1997, a ribbed, secretion case; and *Orthotrichia tyleri* Wells, 1997, a case that is almost identical in shape to the case of *Caledonotrichia extensa*. These three species have been collected from northern Australian billabongs (anabranchs) and streams, all of which have very warm waters where oxygen levels might be expected to be low at times. The vents may be an adaptation that, together with undulations of the body, assists water circulation in the case and thus improve ventilation. Small dorsal openings are seen also in the dome-shaped cases of *Caledonotrichia illiesi* (Wells 1995: figs 11, 12), possibly serving a similar function for their inhabitants.

Sorting of *Caledonotrichia* adults is fraught. Females are generally very closely similar in appearance and usually we have made little attempt to sort them. To illustrate general form we figure and describe several that have been associated tentatively. Males of most species are difficult to identify, too, without close scrutiny of individual specimens under a compound microscope; this is not feasible for sizeable samples. However, on the basis of morphology of male genitalia the species fall into two distinct species groups — an ‘*illiesi* group’ and an ‘*extensa* group’. The three ‘*extensa* group’ species are relatively easy to identify when in alcohol. The males all have abdominal segment IX strongly triangular in shape and *Caledonotrichia extensa* is the largest of all congeners. *Caledonotrichia nyurga* and *Caledonotrichia sykorai* have elongate processes apico-mesally on abdominal sternite VIII, and *Caledonotrichia nyurga* has antennae with distinctive rounded to urn-shaped distal segments. Identifying males of the other group is more difficult — mainly because the genitalia are very hairy and often withdrawn into abdominal segment IX, and in preserved specimens the two lobes of the gonopods are usually folded tightly towards the body, obscuring all other genitalic structures. The only way to identify them with certainty is by macerating the genitalia. Males of four species, however, can be identified by the scales on the wings, although these may be deciduous or simply lost due to abrasion when collected. *Caledonotrichia minor*, *Caledonotrichia ouinnica* sp. n. and *Caledonotrichia capensis* sp. n. all have scales on the forewing only; *Caledonotrichia charadra* has them on both fore- and hind wings. The extent of the scale patches can be used to separate these first three listed species. The scales are possibly androconial scales, involved in scent dispersal and in the male lekking behaviour was observed in the field in this group that appear to be primarily diurnal in behaviour. Specimens of several specieswere collected (by AW) in bright sunlight as they (predominantly males) rested, ran or flew around on emergent rocks or on riparian vegetation, usually in small groups. Similar diurnal behaviour is exhibited by some species in the Stactobiinae genera *Chrysotrichia* Schmid, 1958 and *Scelotrichia* Ulmer, 1951, and some of these have scales on their wings (Wells & Huisman 1993).

The peculiarly diverse case forms seen in *Caledonotrichia* are more or less paralleled in the Stactobiinae, which share with *Caledonotrichia* features such as head with tentorium complete, mesoscutellum with transverse suture and, on the forewing, a well-developed jugal lobe, features not noted by Marshall (1979), possibly because they are probably plesiomorphic.

Recent authors such as Morse (2012) and Holzenthal et al. (2007), when considering Hydroptilidae relationships or classification, have also failed to place *Caledonotrichia*, leaving it in *incertae sedis* in family Hydroptilidae. A sister group relationship between *Caledonotrichia* and the Australian *Maydenoptila* was postulated by Wells (1995). Both have the above plesiomorphic features and their males have bilobed gonopods, with one lobe of the pair with a mesal process of some kind; in both the phallic apparatus varies in form between species, some with one or more associated parameres, others simple; and most species of *Maydenoptila* and at least three of *Caledonotrichia* have abdominal segment IX strongly triangular in ventral view. Females in both genera have similar slender, elongate abdominal terminalia. Although *Caledonotrichia* and *Maydenoptila* share many features, they exhibit some notable differences. The wings of *Caledonotrichia* are narrower than those of *Maydenoptila*, with the venation considerably reduced. No known species of *Maydenoptila* is modified in this way; indeed, most have wings that are somewhat broader than those of many Hydroptilinae, and with venation more complete than most. Another notable difference is the occurrence of scent scales or androconia on wings of males of some *Caledonotrichia*; these are not known to occur speciesof *Maydenoptila*. Evolution of scales on wings may be a phenomenon that sometimes occurs when species diverge in sympatry such as may be the situation from time to time on islands — wing scales are found in a Lord Howe Island species of *Orphninotrichia* Mosely, but not in any of the Australian mainland species (Wells 1999, 2010); an *Oxyethira* species with scattered scales on the hind wing was described by Johanson et al. (2011) from the island of Espiritu Santo, Vanuatu. Wing scales occur also in stactobiine taxa such as *Chrysotrichia* and on wings of a number of Neotropical leucotrichiine species, too.

The distribution of *Maydenoptila* in south-western and eastern Australia suggests that it could be Gondwanan in origin and *Caledonotrichia* could have a similar origin. Thus, if *Caledonotrichia* and *Maydenoptila* are members of the Stactobiinae, they probably evolved from an early stactobiine lineage. Note, however, that contrary interpretations are given by Harris & Armitage (1997) who, in their study of the Neotropical genus *Nothotrichia* Flint, 1967 concurred with Kelley (1992) in placing that genus with *Caledonotrichia* and *Maydenoptila* in the basically Neotropical ‘tribe [sic] Ochrotrichiinae’. Relationships of these genera remain to be tested by studies based on molecular data.

In support of future studies on New Caledonian Hydroptilidae, we provide diagnoses and descriptions for all known species of *Caledonotrichia*, along with an identification key to adult males. Other hydroptilid genera found in New Caledonian freshwater systems — *Paroxyethira*, *Hellyethira*, *Acritoptila* and *Oxyethira* — are (Wells & Johanson 2012), or will be, treated elsewhere.

## Material and methods

Adult specimens were collected in light traps and Malaise traps situated near running waters, swept from riparian vegetation or from emergent boulders and cobbles in streams, or ‘dabbed’ using an alcohol-dipped finger tip. Specimens were prepared for study as Canada balsam slide mounts following the methods of Wells (1980). Male genitalia are illustrated in line drawings and also, for species for which suitable slides are available, as images derived using the digital imaging software AutoMontage. This duplication of effort allow readers to understand the morphology of the male genitalia of *Caledonotrichia* species and will aid identifications. Descriptions are based primarily on males. Specimens in this study are deposited in the following repositories:

**MNHP** Muséum National d’Histoire Naturelle, Paris, France

**NHRS** Naturhistoriska riksmuseet, Stockholm, Sweden

**ANIC** Australian National Insect Collection, CSIRO Ecosystem Sciences, Canberra, Australia

**QM** Queensland Museum, Brisbane, Australia

**ROM** Royal Ontario Museum.

**BPBM** Bishop Museum, Hawaii, USA

## Descriptions

### Caledonotrichia

Sykora

http://species-id.net/wiki/Caledonotrichia

Caledonotrichia Sykora (1967: 585); Marshall (1979: 221); Kelley (1989: 194); Wells (1995: 224).

#### Type species.

*Caledonotrichia illiesi* Sykora, by original designation.

#### Revised description, male.

Head wider than long, in dorsal view variably rounded to subrectangular; 3 ocelli present; antennae with 22–37 flagellomeres in male, 20–24 flagellomeres in female; tentorium complete, posterior bridge well developed, dorsal arms vestigial; maxillary palps with 2 basal and fourth segments short, other segments elongate; clypeus bearing a dense brush of setae. Forewing length 1.0–3.5 mm, broad to narrowly acuminate, with or without patches of specialised scent scales (androconial scales); jugal lobe present; hind wing with or without scales; venation of both wings modified to a greater or lesser extent, width of wings variable, generally slender with apices acuminate. On thorax, mesoscutellum with transverse suture; metascutellum triangular. Tibial spur formula 0,3,4. Female terminalia forming slender, elongate oviscapt. Male genitalia with abdominal segment IX well developed, broadly to narrowly shield-shaped, or triangular in ventral view and triangular in lateral view. Tergite X membranous, short, longer than wide. Gonopods bilobed, dorsal lobe irregularly elongate, subquadrate to rectangular or rounded, usually longer than ventral lobe, usually bearing a digitiform mesal process, ventral lobe triangular to bean-shaped, or narrowly leaf-shaped; in axil between dorsal and ventral lobe usually a small, rounded, setate process and basally on ventral lobe, a strong elongate seta. Subgenital processes (or plate) in form of pair of sclerotised rods which, in lateral aspect, strongly sinuate. Phallic apparatus elongate, with or without associated parameres.

**Figures 1–10.**
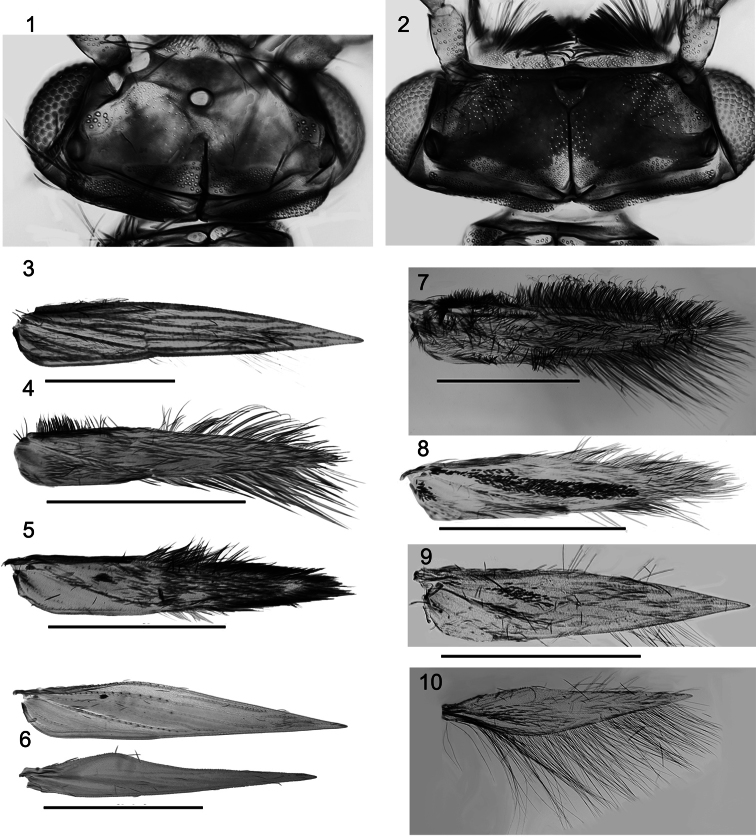
Male *Caledonotrichia* head and wings from Automontage photo. **1** Head of *Caledonotrichia capensis* sp. n. dorsal view **2** Head of *Caledonotrichia vexilla* sp. n. dorsal view **3** *Caledonotrichia extensa* Kelley right forewing **4** *Caledonotrichia minuta* right forewing **5** *Caledonotrichia minor* Sykora right forewing **6** *Caledonotrichia ouinnica* sp. n. right fore and hind wing **7** *Caledonotrichia vexilla* sp. n. right forewing **8** *Caledonotrichia capensis* sp. n, right forewing **9** *Caledonotrichia charadra* Kelley right forewing **10** *Caledonotrichia charadra* Kelley right hind wing. Scale bars = 1.0 mm.

**Figures 11–20.**
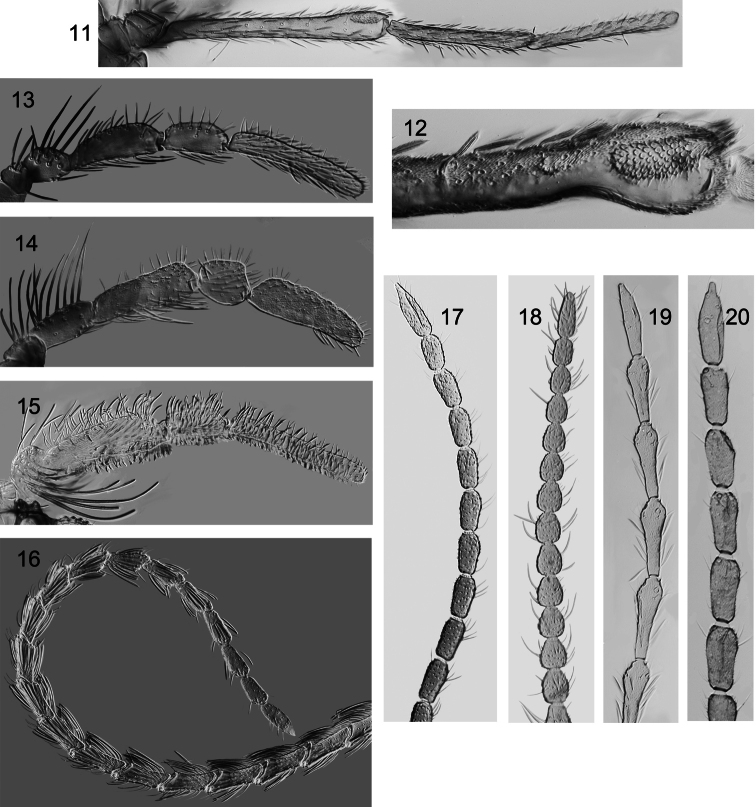
Male *Caledonotrichia* maxillary palp and distal part of antenna from Automontage photo. **11** *Caledonotrichia extensa* Kelley maxillary palp ventral view **12** male maxillary palp ventral view showing area of sensilla on segment 3 **13** *Caledonotrichia minuta* sp. n. maxillary palp ventral view **14** *Caledonotrichia bifida* sp. n. maxillary palp ventral view **15** *Caledonotrichia ouinnica*, sp. n. maxillary palp ventral view **16** *Caledonotrichia bifida*, sp. n. antennae **17**, *Caledonotrichia sykorai*, sp. n. antenna **18** *Caledonotrichia nyurga* Oláh & Johanson antenna **19** *Caledonotrichia extensa* Kelley antenna **20** *Caledonotrichia illiesi* Sykora antenna.

**Figures 21–29.**
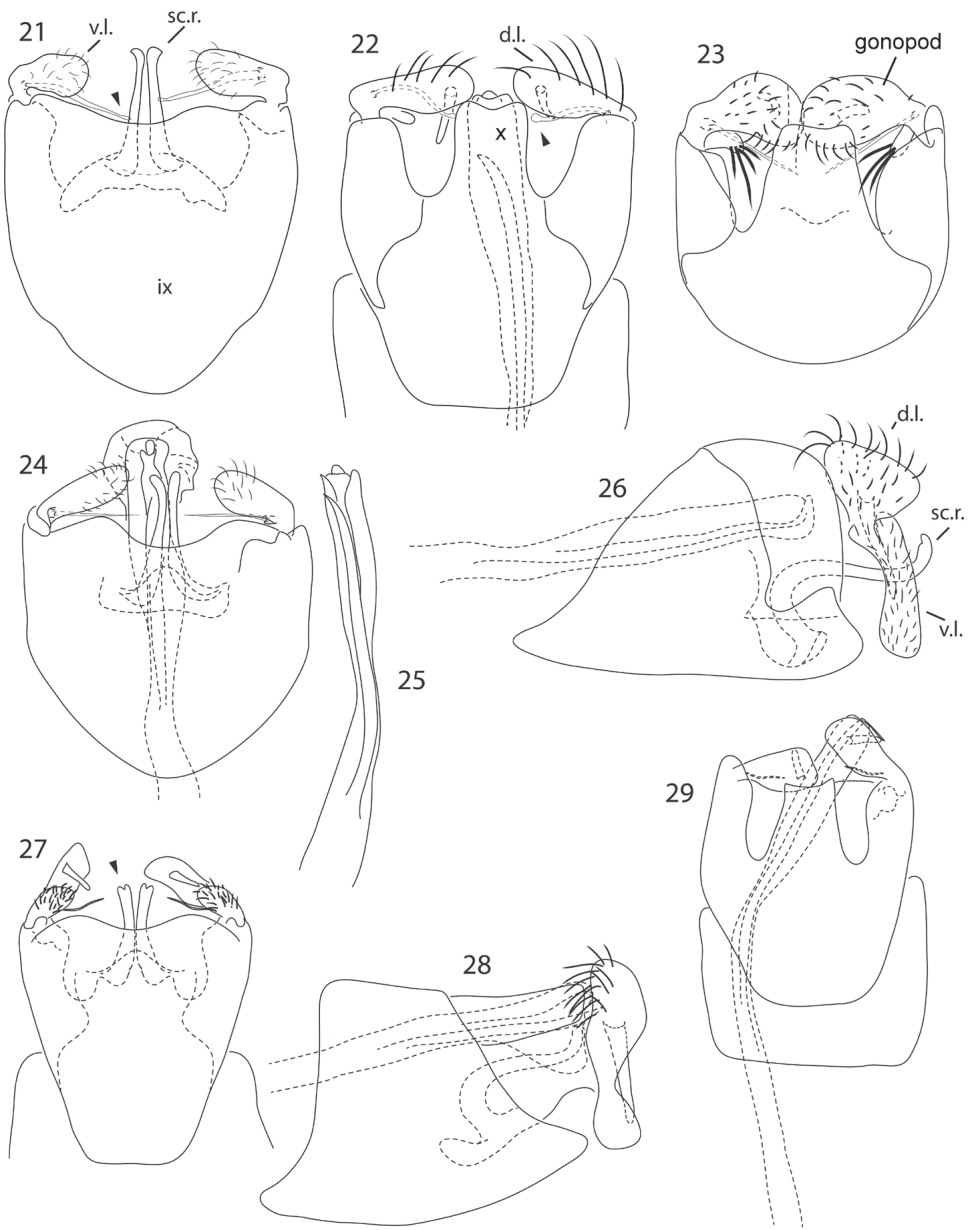
*Caledonotrichia*, male genitalia. **21** *Caledonotrichia illiesi* Sykora ventral view **22** *Caledonotrichia illiesi* Sykora dorsal view **23** *Caledonotrichia minuta* sp. n. dorsal view **24** *Caledonotrichia minuta* sp. n. ventral view **25** *Caledonotrichia minuta* sp. n. phallic apparatus dorsal view **26** *Caledonotrichia minuta* sp. n. lateral view **27** *Caledonotrichia bifida*, sp. n. ventral view **28** *Caledonotrichia bifida*, sp. n. lateral view **29** *Caledonotrichia bifida* sp. n. dorsal view. Abbreviations: ix = segment IX, x = tergite X, v.l. = ventral lobe of gonopod, d.l. = dorsal lobe of gonopod, s.r. = sclerotised rod of subgenital process.

**Figures 30–35.**
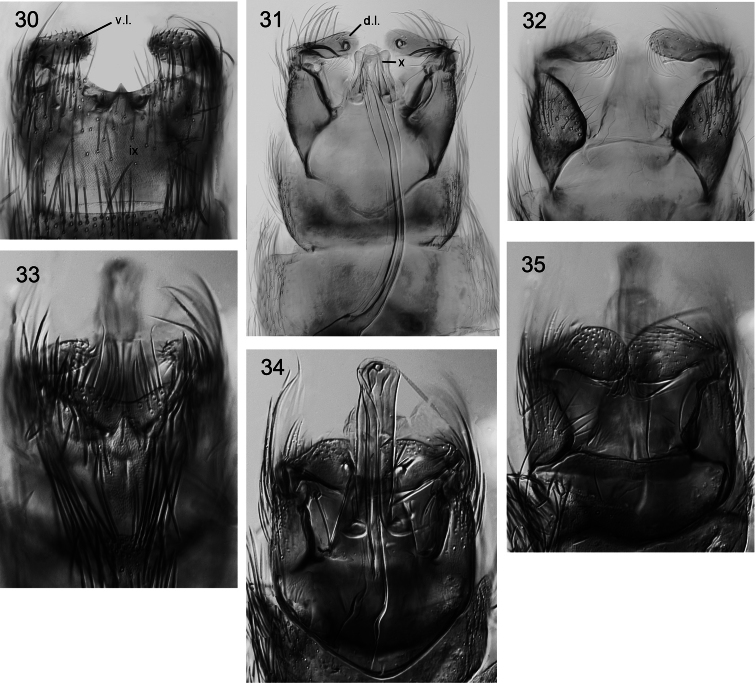
*Caledonotrichia*, male genitalia from Automontage photo. **30** *Caledonotrichia illiesi* Sykora ventral view **31** *Caledonotrichia illiesi* Sykora ventral side of dorsal lobes of gonopods **32** *Caledonotrichia illiesi* Sykora dorsal view **33** *Caledonotrichia minuta* sp. n. ventral view **34** *Caledonotrichia minuta* sp. n. ventral view of ventral side of dorsal lobes of gonopods **35** *Caledonotrichia minuta* sp. n. dorsal view. Abbreviations: ix = segment IX, x = tergite X, v.l. = ventral lobe of gonopod, d.l. = dorsal lobe of gonopod.

**Figures 36–43.**
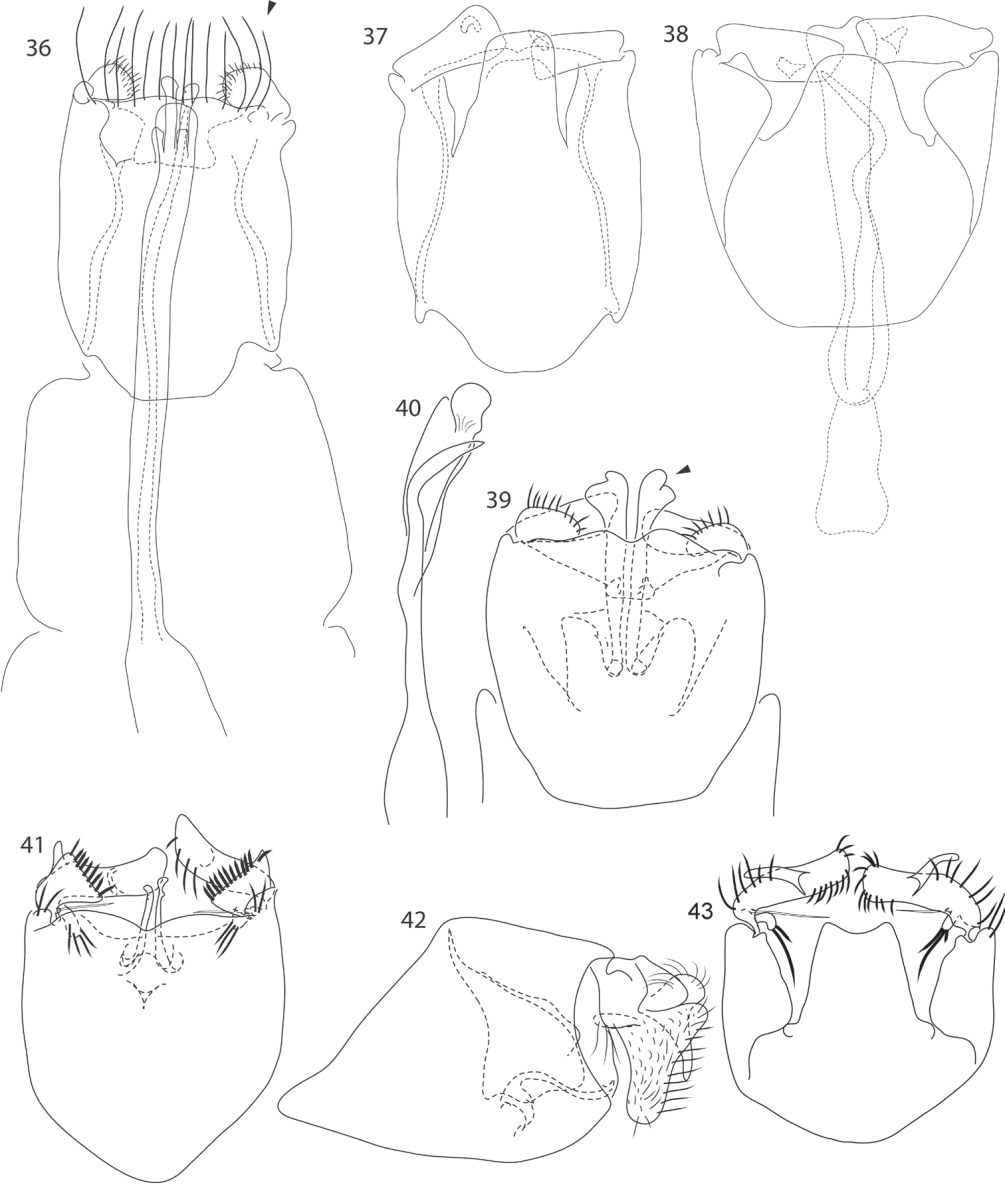
*Caledonotrichia*, male genitalia. **36** *Caledonotrichia ouinnica*, sp. n. ventral view **37** *Caledonotrichia ouinnica*, sp. n. dorsal view **38** *Caledonotrichia minor* Sykora dorsal view **39** *Caledonotrichia minor* Sykora ventral view **40** *Caledonotrichia minor* Sykora phallic apparatus ventral view **41** *Caledonotrichia vexilla* sp. n. ventral view **42** *Caledonotrichia vexilla* sp. n. lateral view **43** *Caledonotrichia vexilla* sp. n. dorsal view.

**Figures 44–50.**
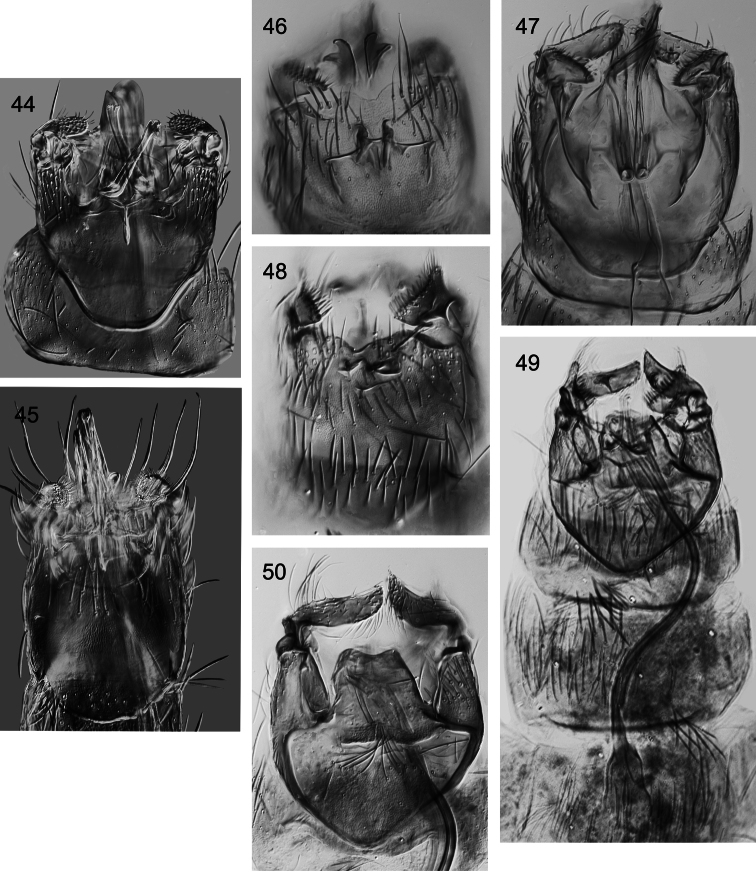
*Caledonotrichia*, male genitalia from Automontage photo. **44** *Caledonotrichia bifida* sp. n. ventral view **45** *Caledonotrichia ouinnica* sp. n. ventral view **46** *Caledonotrichia minor* Sykora ventral view **47** *Caledonotrichia minor* Sykora dorsal view **48** *Caledonotrichia vexilla* sp. n. ventral view **49** *Caledonotrichia vexilla* sp. n. ventral side of dorsal lobes of gonopods **50** *Caledonotrichia vexilla* sp. n. dorsal view.

**Figures 51–56.**
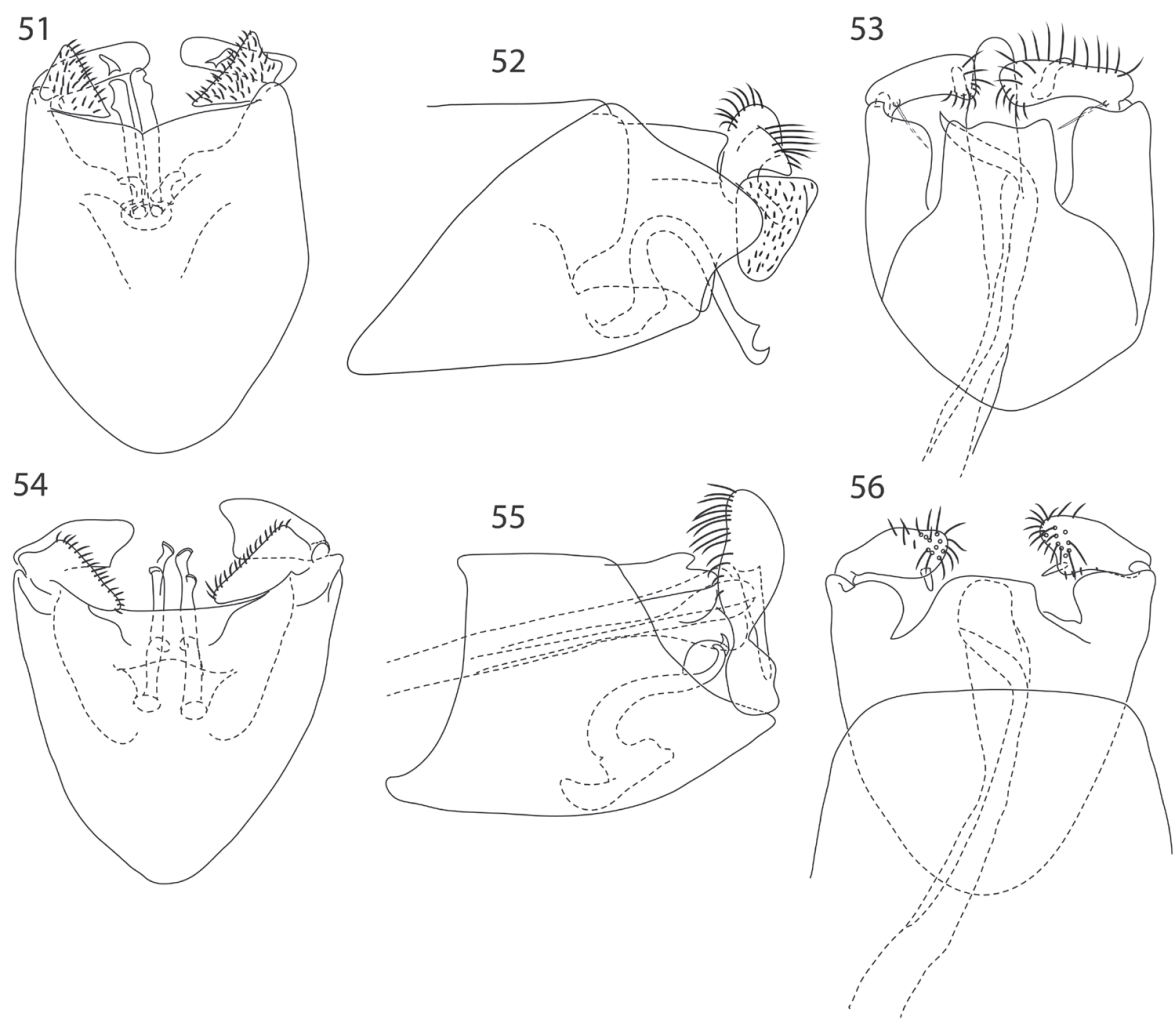
*Caledonotrichia*, male genitalia. **51** *Caledonotrichia capensis* sp. n. ventral view **52** *Caledonotrichia capensis* sp. n. lateral view **53** *Caledonotrichia capensis* sp. n. dorsal view**54** *Caledonotrichia charadra* Kelley ventral view **55** *Caledonotrichia charadra* Kelley lateral view **56** *Caledonotrichia charadra* Kelley dorsal view.

**Figures 57–63.**
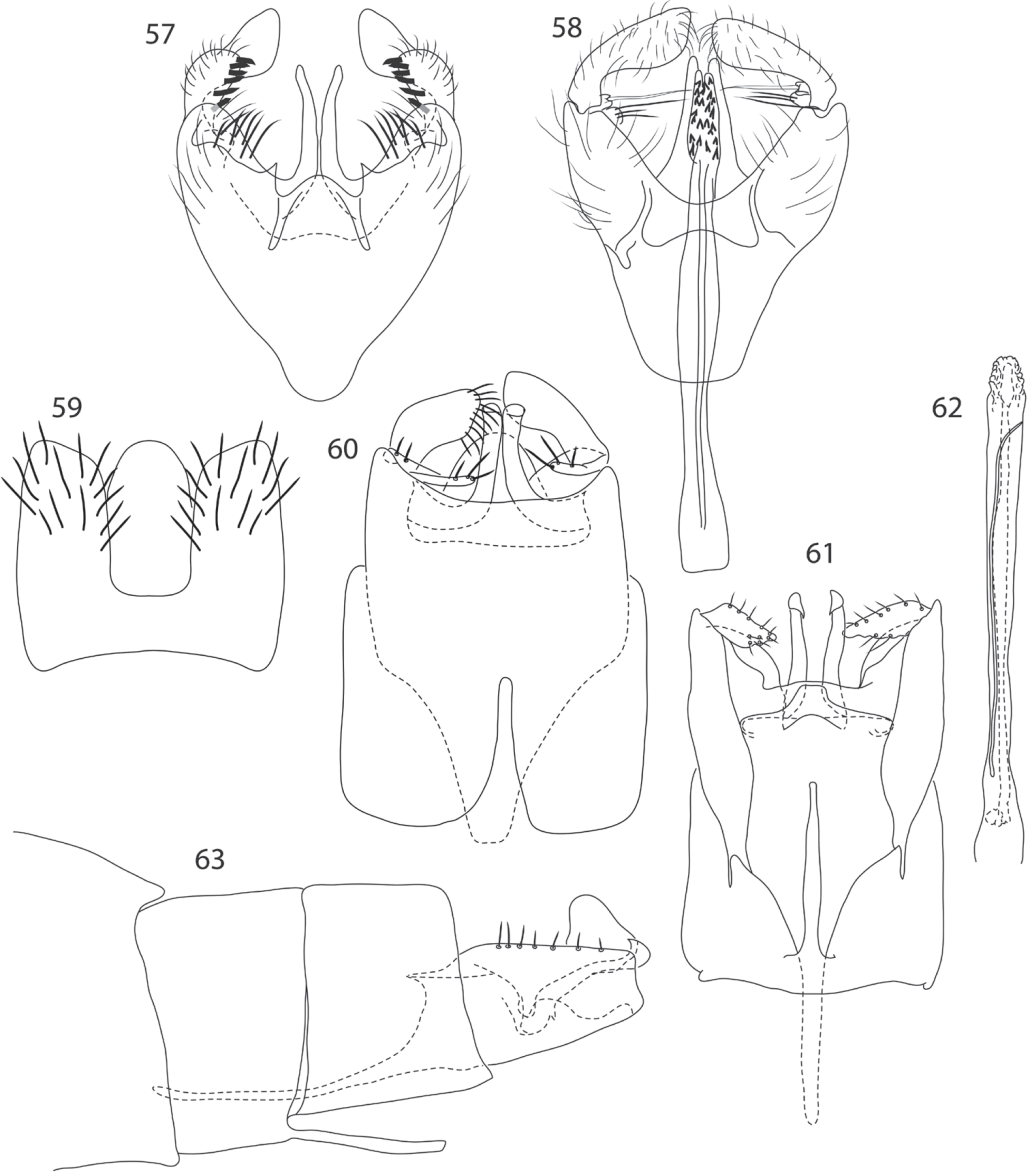
*Caledonotrichia*, male genitalia. **57** *Caledonotrichia extensa* Sykora ventral view **58** *Caledonotrichia extensa* Sykora dorsal view **59** *Caledonotrichia sykorai* sp. n. dorsal view **60** *Caledonotrichia sykorai* sp. n. ventral view **61** *Caledonotrichia nyurga* Oláh & Johanson ventral view **62** *Caledonotrichia nyurga* Oláh & Johanson phallic apparatus ventral view **63** *Caledonotrichia nyurga* Oláh & Johanson lateral view.

**Figures 64–70.**
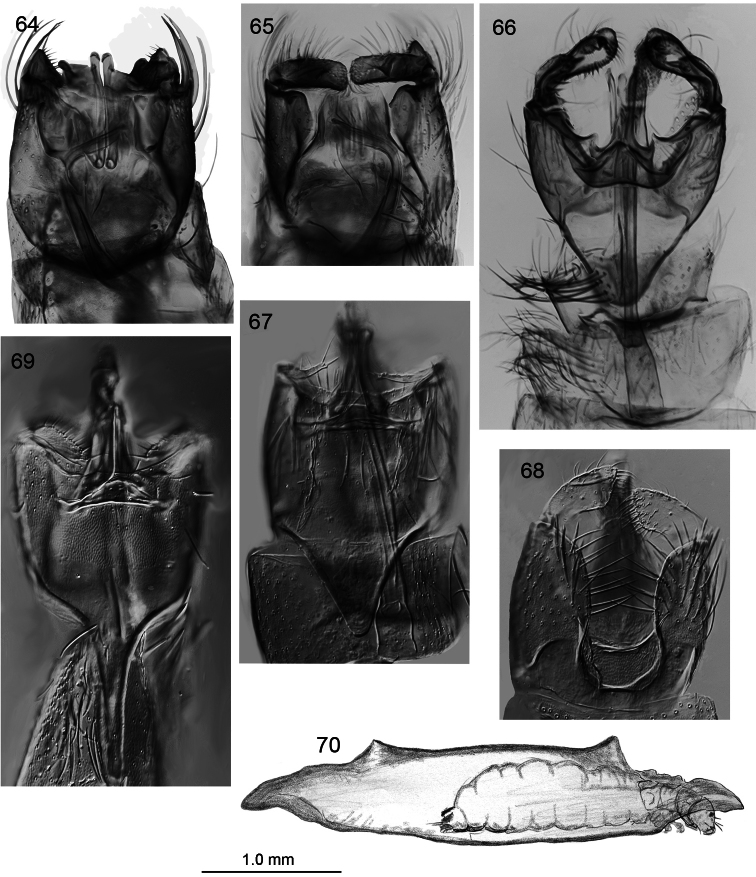
*Caledonotrichia*, male genitalia from Automontage. **64** *Caledonotrichia capensis* sp. n. ventral view **65** *Caledonotrichia capensis* sp. n. dorsal view **66** *Caledonotrichia extensa* Kelley ventral side of dorsal lobes of gonopods **67** *Caledonotrichia sykorai* sp. n. ventral view **68** *Caledonotrichia sykorai* sp. n. dorsal view **69** *Caledonotrichia nyurga* Oláh & Johanson dorsal view **70** *Caledonotrichia extensa* Kelley final instar larva in case in lateral view.

**Figures 71–73.**
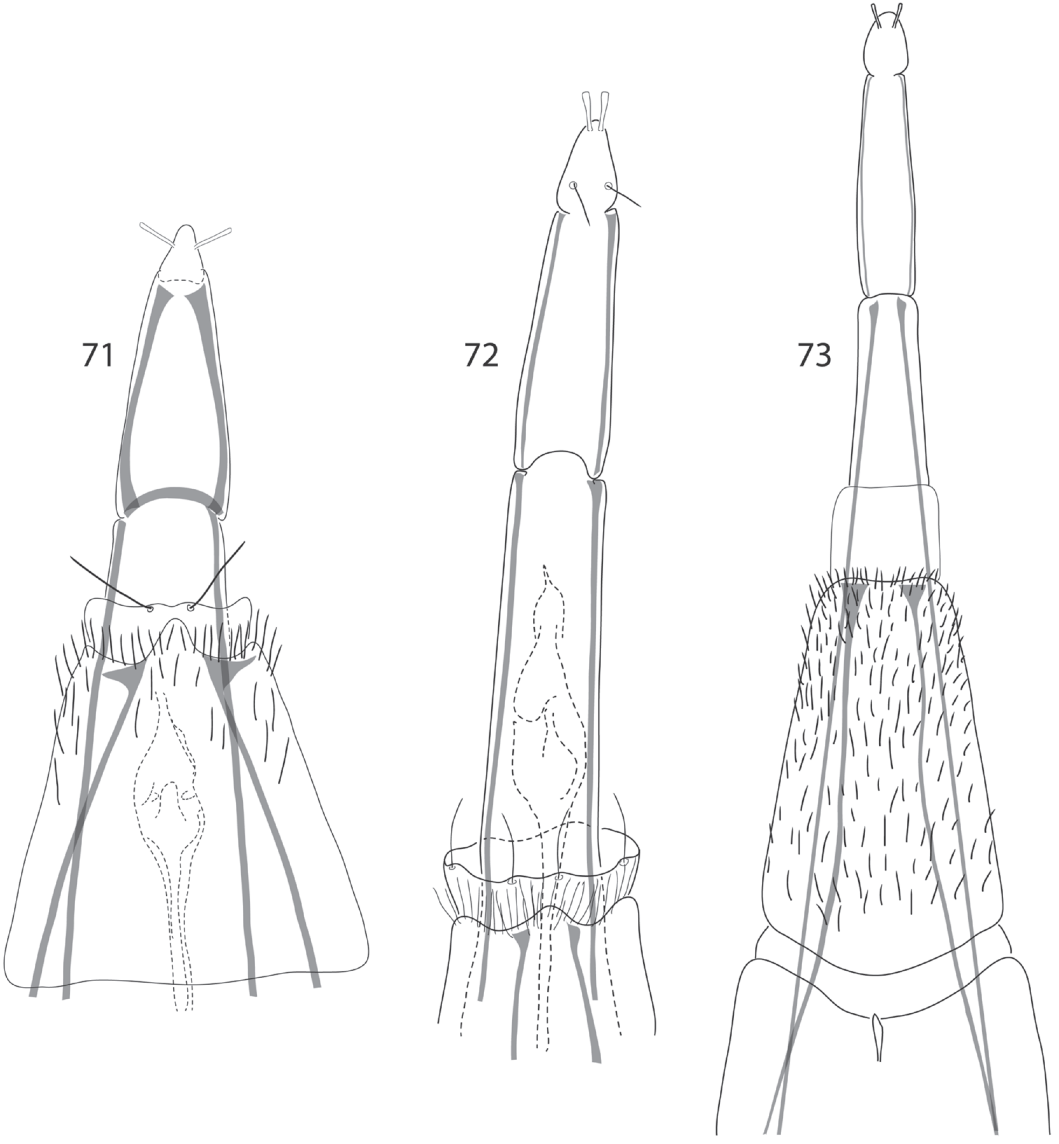
*Caledonotrichia*, female terminalia. **71** *Caledonotrichia illiesi* Sykora ventral view **72** *Caledonotrichia minuta*, sp. n. ventral view **73** *Caledonotrichia sykorai* sp. n. ventral view.

#### Larva.

Diagnoses are given for larvae by Marshall (1979) and Wells (1995: 228). Mature larvae are basically plesiomorphic, with or without dorsal abdominal sclerites; cases highly variable in shape and materials.

### Caledonotrichia illiesi

Sykora

http://species-id.net/wiki/Caledonotrichia_illiesi

Figs 20 22 30–32 71

Caledonotrichia illiesi Sykora (1967: 585–595); Wells (1995: 229).

#### Revised diagnosis.

In many respects males of *Caledonotrichia illiesi* resemble the smaller *Caledonotrichia minuta* sp. n., and *Caledonotrichia bifida* sp. n., having wings without scales and both lobes of gonopods are rounded, but are readily recognized by their large size, more robust in appearance, have thick brushes of short black setae dorsally on head and have elongate antennae about equal in length to body. In the male genitalia (Figs 21–22, 30–32) the subgenital process forms simple, straight sclerotised rods, and the elongate seta in the axil between the upper and lower lobes of each gonopod terminates in a round apical knob (arrow in Figs 21, 22). In the female terminalia (Fig. 71) abdominal sternite VII has a triangular prominence apico-medially, and a membranous collar; segment X is triangular.

#### Revised description, male.

Head rounded in dorsal view, as in *Caledonotrichia capensis* sp. n. (Fig. 1). Antennae with 35–37 flagellomeres; flagellomeres (Fig. 20) elongate cylindrical, length about 2× width. Maxillary palps with basal 2 segments short and rounded, rest cylindrical: segment 3 about 4× maximum width, segment 4 length about 2× width, and segment 5 elongate slender, length almost 6n width. Forewing length, 2.1–3.0 mm (n=10). Wings without scales, forewing costa with brush of straight setae on proximal third, then longer, curved setae to tip of wing.

#### Additional information, female.

Antennae with 33 flagellomeres. Forewing length, 2.0–3.2 mm (n=10).

#### Material examined.

Holotype male: New Caledonia: River near Col d’Amieu (BPBM), examined. 1 pharate male pupa, upper Hienghène R. at Kavatch, 6.ix.1965, F. Starmühlner (ROM); 1 pharate male pupa, tributary Hienghène R. at Castex Station 5 km below Kavatch, 6.viii.1965, F. Starmühlner (ROM); 1 male, creek at end of Col de Petchékara, 19.xii.1983, A. Wells (ANIC); 3 males, larvae and pupae, Bopope, 18.xii.1983, A. Wells, ANIC; 1 larva, Ck between Négropa and Koh on La Foa-Canal Road, 19.xii.1983,A. Wells (ANIC); 2 males, Forêt Thy Reserve, 150 m, 21.v.1984, G. Monteith, D. Cook, QM; 2 males, 1 female, stream beside Farino road, 20.xii.1998, A. Wells, ANIC; 2 males, approx 15 km SW of Houailou on Houailou-Bourail road, small fall, 26.xii.1998, A. Wells (ANIC); 3 males, ~10 km NW Hienghène, small stream, 25.xii.1998, A. Wells (ANIC); 4 males, 2 females Rivière du Cap, Pont du Cap, ~8 km NW Naindai on Bourail-Poya road, 22.xii.1998, A. Wells (ANIC); 11 males, 12 females, stream approx. 20 km SW Thio on Boulouparis-Thio road, 28.xii. 1998, A. Wells (ANIC); 2 males, UFP-LERVEM, Tay 2, 18.x.1999, N. Mary (ANIC); 24 males, 17 females, Province Sud, Monts Kwa Ne Mwa, on road between Noumea and Yaté, Rivière des Pirogues, 22°11.225'S, 166°43.338'E, 100 m, 7.xi.2003, light trap, loc#016, K. A. Johanson (NHRS); numerous males & females, Province Sud, side stream to Rivière Blanche, 10.75 km SW Pont Pérignon, 22°10.073'S, 166°39.903'E, 180 m, 6–16.xi.2003, Malaise trap, loc#012, K. A. Johanson (NHRS); 15 males, 8 females, Province Sud, Mt. Dzumac, source stream of Ouinne River, near crosspoint to mountain track, 22°02.439'S, 166°28.646'E, 805 m, 18.xi–4.xii.2003, Malaise trap, loc#029, K. A. Johanson (NHRS); 7 males, 4 females, Province Sud, Mt. Dzumac, source stream of Ouinne River, near crosspoint to mountain track, 22°02.073'S, 166°28.460'E, 810 m, 18.xi–4.xii.2003, Malaise trap, loc#030, K. A. Johanson (NHRS); 1 male, on slide, Province Sud, Mt. Dzumac, source stream of Ouinne River, downstream crosspoint to mountain track, 22°01.997'S, 166°28.486'E, 795 m, over about 30 m waterfall, 18.xi–4.xii.2003, Malaise trap, loc#031, K. A. Johanson (NHRS); 13 males, 8 females, Province Sud, W slope Mt. Ningua, Kwé Néco Stream, at Camp Jacob, 3.9 km W summit of Mt. Ningua, on Boulouparis–Thio Road, about 50 m upstream road, 21°44.083'S, 166°06.298'E, 117 m, 29.xi–12.xii.2003, Malaise trap, loc#053, K. A. Johanson (NHRS); numerous males, females, Province Sud, W slope Mt. Ningua, Kwé Néco Stream, 3.9 km W summit of Mt. Ningua, on Boulouparis–Thio Road, about 50 m upstream road, 21°44.359'S, 166°06.009'E, 117 m, 20.xi–12.xii.2003, Malaise trap, loc#035, K. A. Johanson (NHRS); males, females, Province Sud, W slope Mt. Ningua, Kwé Néco, Stream, at Camp Jacob, 3.7 km WNW summit of Mt. Ningua, on Boulouparis–Thio Road, about 50 m upstream road, 21°43.613'S, 166°06.567'E, 150 m, 29.xi–12.xii.2003, Malaise trap, loc#054, K. A. Johanson (NHRS); 6 males, Province Sud, Mt Rembai, River Xwâ Be, upstream bridge on road Sarraméa-Koh, 21°33.877'S, 165°49.922'E, loc 157F-k, Malaise trap, 8.vii–4.viii.2007, R. Pöllabauer (NHRS); 6 males, loc 157 F-K, 8.vii–4.viii.2007; 1 male, New Caledonia, Chute S of Col d’Amieu on Sarraméa-Thio road, 2.iv.2012, A. Wells & S. Cazères (ANIC); 7 males, 1 female, Chute de Farina, ~5 km N of Farino, 15.iv.2012, A. Wells (ANIC).

### Caledonotrichia minuta sp. n.

urn:lsid:zoobank.org:act:383499C5-C46C-4F4A-99B7-DD93A865AA64

http://species-id.net/wiki/Caledonotrichia_minuta

Figs 4 13 23–26 33–35 72

#### Diagnosis.

Males very closely resemble *Caledonotrichia illiesi* and *Caledonotrichia bifida* sp. n., both of which have wings without scales and both lobes of gonopods rounded, but *Caledonotrichia minuta* differs from *Caledonotrichia illiesi* by smaller size and far less robust appearance, antennae shorter than wings, and in male genitalia dorsal lobe of gonopods more broadly rounded and bat-shaped, and from *Caledonotrichia bifida* sp. n. by having ventral lobes of gonopods almost equal length to dorsal lobes, dorsal lobes more broadly rounded, and sclerotised rods of subgenital process with only small irregularity subapically, not bifid apically as in *Caledonotrichia bifida* sp. n.

#### Description, male.

Head rounded in dorsal view, as in *Caledonotrichia capensis* sp. n. (Fig. 1).

Antennae, male with 23–24 flagellomeres, female with 22 flagellomeres; in male, proximal flagellomeres elongate cylindrical, length not more than 2× width, more distal flagellomeres subquadrate. Male maxillary palps (Fig. 13) similar to those of *Caledonotrichia illiesi*, with basal 2 segments short and rounded, rest cylindrical: segment 3 about 4× maximum width, segment 4 length about 2× width, segment 5 elongate, length almost 6× width. Forewing length, male 1.5–2.2 mm (n=10); female 2.0–2.5 mm (n=10). Male forewing (Fig. 4) without scales, narrow, apically acute, with upright bristles on costal vein, hair on distal two thirds of costal margin with strongly curved tips as in *Caledonotrichia illiesi*.

Genitalia (Figs 23–26, 33–35): Abdominal segment IX broadly rounded proximally, distal margin of sternite shallowly excavated medially. Tergite X, tapered slightly to broad, slightly concave apex. Gonopods in ventral view with ventral lobes elongate club-shaped, dorsal lobes broadly rounded, mesal process digitiform, well developed, without setae; axillary seta well developed, acute apically. Sclerotised rods of subgenital process, simple, a slight irregularity below apex. Phallic apparatus elongate, almost straight, broadly rounded distally, with a slender almost straight spiny paramere.

Female terminalia (Fig. 72). Abdominal segment VII bearing fringe of dense short setae distally and apically a short, membranous collar bearing sparsely arranged setae marginally, sternite with a small medial prominence apically; abdominal segments VIII–X forming slender telescopic oviscapt.

#### Material examined.

Holotype male: New Caledonia: approx 10 km SW of Houailou on Houailou-Bourail road, small fall, 26.xii.1998, A. Wells (MNHP).

Paratypes: 10 males, same data as for holotype (ANIC); 4 males, approx 10 km NW Hienghène, small stream, 25.xii.1998, A. Wells (ANIC); 3 males, approx 15 km SW Thio on Boulouparis-Thio road, 28.xii.1998, A. Wells (ANIC); 1 male, 1 female (on slides), Province Sud, Mt. Dzumac, source stream of Ouinne River, near crosspoint to mountain track, 22°02.073’S, 166°28.460’E, 810 m, 18.xi–4.xii.2003, Malaise trap, loc#030, K. A. Johanson (SMNH); 11 males, Province Sud, Mt Rembai, River Xwâ Be, upstream bridge on road Sarraméa-Koh, 21°33.877’S, 165°49.922’E, loc 157F-k, Malaise trap, 8.vii–4.viii.2007, R. Pöllabauer (SMNH); 28 males, Chute S of Col d’Amieu on Sarraméa-Thio road, 2.iv.2012, A. Wells & S. Cazères (ANIC).

Other material examined: 1 male, Chute approx 15 km N Col d’Amieu on Boulouparis-Thio road, 27.xi.1998, A. Wells, ANIC; 1 male, Province Sud, stony stream draining Lac Yaté, 200 m, loc 5, 22°08.795'S, 166°42.313'E, Malaise trap 13–16.xi.2001, Johanson, Pape & Viklund (NHRS); 9 males, 14 females, Province Sud, Sarraméa, 290 m, stony forest stream, loc 13 21°37.097'S 165°49.351'E, Malaise trap, 18–21.xi.2001, Johanson, Pape & Viklund (NHRS); 55 males, 18 females, Province Sud, Col d'Amieu, 323 m, small stony river, loc 24, 21°34.844'S, 165°49.677'E, Malaise trap, 30.xi–5.xii.2001, Johanson, Pape & Viklund (NHRS); numerous males, females, Province Sud, Col d'Amieu, 319 m, small stony river, loc 23, 21°34.720'S, 165°49.620'E, Malaise trap, 30.xi–5.xii.2001, Johanson, Pape & Viklund (NHRS); numerous males, females, Province Sud, Monts des Koghis, ca 300 m S Koghi Restaurant, 22.18288°S, 166.50167°E, 417 m, 2–16.xi.2003, Malaise trap, loc#004, K. A. Johanson (NHRS); 1 male, Province Sud, stream draining to Marais de la Rivière Blanche, 1.35 km S Pont Pérignon, 22°08.496'S, 166°42.152'E, 180 m, 6–16.xi.2003, Malaise trap, loc#009, K. A. Johanson (NHRS); 8 males, 29 females, Province Sud, stream draining to Marais de la Rivière Blanche, 2.25 km SW Pont Pérignon, 22.14158°S, 166.67993°E, 157 m, 6–16.xi.2003, Malaise trap, loc#010, K. A. Johanson (NHRS); males, females, Province Sud, side stream to Rivière Blanche, 10.75 km SW Pont Pérignon, 22°10.073'S, 166°39.903'E, 180 m, 6–16.xi.2003, Malaise trap, loc#012, K. A. Johanson (NHRS); males, Province Sud, Monts Kwa Ne Mwa, on road between Noumea and Yaté, 2.0 km E Pic Mouirange, 22°12.356'S, 166°40.798'E, 220 m, 7–16.xi.2003, Malaise trap, loc#014, K. A. Johanson (NHRS); 6 males, Province Sud, Monts des Koghis, ca 800 m S Koghi Restaurant, 22.18311°S, 166.50564°E, 460 m, 10–26.xi.2003, Malaise trap, loc#019, K. A. Johanson (NHRS); 1 male, 4 females (male, female on slides), Province Sud, Mt. Dzumac, source stream of Ouinne River, near crosspoint to mountain track, 22°02.073'S, 166°28.460'E, 810 m, 18.xi–4.xii.2003, Malaise trap, loc#030, K. A. Johanson (NHRS); 2 males, 5 females, Province Sud, Monts des Koghis, ca 800 m S Koghi Restaurant, 22.18406°S, 166.50383°E, 420 m, 1126.xi.2003, Malaise trap, loc#022, K. A. Johanson (NHRS); 3 males, 2 females, Province Sud, Hwa Hace Mt., Hwa Motu River, at Pont Wamuttu, 1.0 km E Nassirah, about 200 m upstream bridge, 21°48.094'S, 166°04.298'E, 137 m, 20.xi–12.xii.2003, Malaise trap, loc#034, K. A. Johanson (NHRS); 2 males, 1 female, Province Nord, Wemwâdiu stream, 850 m E summit Kögi Mt., 5 m upstream road, about 200 m S Tiwaka River, 20°49.020'S, 165°14.165'E, 24 m, 6–27.xii.2003, Malaise trap, loc#067, K. A. Johanson (NHRS); 3 males, Province Nord, Wé Caot Stream, draining NNE side of Mt. Panié, 0.9 km NW Cascade de Tao, 20°33.311'S, 164°48.064'E, 18.xii.2003, light trap, loc#084, K. A. Johanson (NHRS); numerous males, females, Province Sud, W slope Mt. Ningua, Kwé Néco Stream, at Camp Jacob, 3.9 km W summit of Mt. Ningua, on Boulouparis–Thio Road, about 50 m upstream road, 21°44.083'S, 166°06.298'E, 117 m, 29.xi.2003–12.xii.2003, Malaise trap, loc#053, K. A. Johanson (NHRS); numerous males, females, Province Nord, Ponandou Tiôgé River at Kögi, 3.9 km SSW Touho, 20°49.043'S, 165°13.551'E, 25 m, 26.xii.2003, light trap, loc#100, K. A. Johanson (NHRS); males, females, Province Sud, W slope Mt. Ningua, Kwé Néco Stream, 3.9 km W summit of Mt. Ningua, on Boulouparis–Thio Road, about 50 m upstream road, 21°44.359'S, 166°06.009'E, 117 m, 20.xi–12.xii.2003, Malaise trap, loc#035, K. A. Johanson (NHRS); 3 males, Province Nord, 50 m upstream bridge on Hienghène-Tnèdo road, 3.9 km S summit of Mt. Tnèda, 2.2 km E Tnèdo, 20°43.085'S, 164°49.928'E, 29 m, 7.xii.2003, light trap, loc#071, K. A. Johanson (NHRS); 6 males, 15 females, Province Sud, stream crossing way to sanatorium 2.3 km E St Laurent, ca. 150 m upstream bridge, 22°04.484'S, 166°19.910'E, loc 027, Malaise trap, 17–19.x.2006, K. A. Johanson & M. Espeland (NHRS); 2 males, 2 females, Province Sud, stream crossing way to sanatorium 2.3 km E St Laurent, ca. 150 m upstream bridge, 22°04.484'S, 166°19.910'E, loc 027, Malaise trap, 17–19.x.2006, K. A. Johanson & M. Espeland (NHRS); 11 males, numerous females, Province Nord, Ponandou Tiôgé River at Kögi, 3.9 km SSW Touho, 20°49.043'S, 165°13.551'E, 25 m, 26.xii.2003, light trap, loc#100, K. A. Johanson (NHRS).

**Figure 74.**
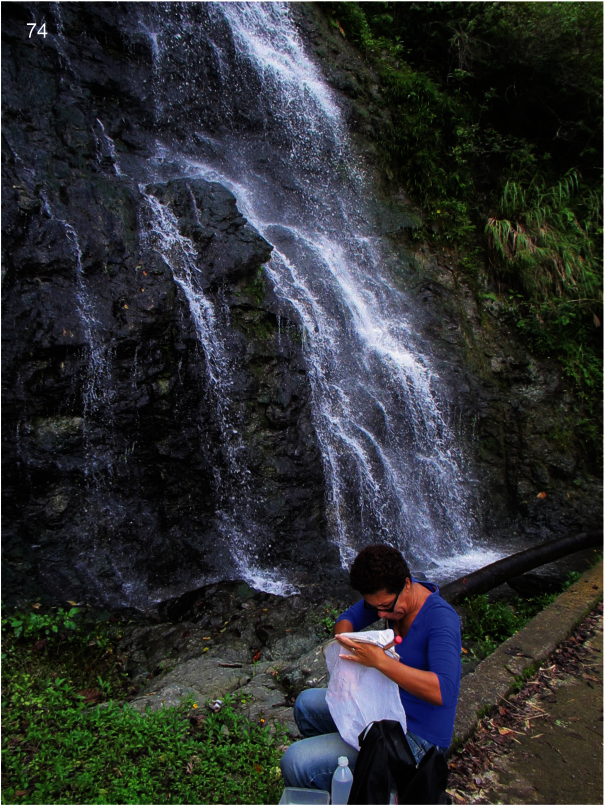
Steep waterfall at Col d’Amieu, Southern Province, New Caledonia, 2.iv.2012, where adults of *Caledonotrichia minuta* sp. n. were collected by Sylvie Cazères (on photo) using a sweep-net and aspirator.

#### Etymology.

*Minuta*, name referring to the small size of the species.

#### Remarks.

This species appears to be widespread and often abundant but, like most of the *Caledonotrichia* species, is hard to identify with certainty unless it is mounted on a microscope slide. Thus, only a small number of specimens are designated as paratypes. Considerable numbers of these tiny mostly jet black caddisflies, with shiny silver areas of setae on their wings, most of them males, were seen running around in bright sunlight on exposed rocks at waterfalls. Fig. 74 shows a locality where a large number of specimens, mostly males, of this species were collected by sweep net.

### Caledonotrichia bifida sp. n.

urn:lsid:zoobank.org:act:66E69363-3948-43E6-8920-23136A78D4AA

http://species-id.net/wiki/Caledonotrichia_bifida

Figs 14, 16 27–29 44

#### Diagnosis.

Males very closely resembling *Caledonotrichia illiesi* and *Caledonotrichia minuta* sp. n., with which they share the features of wings without scales and both lobes of gonopods rounded. Like *Caledonotrichia minuta*, they differ from *Caledonotrichia illiesi* by their smaller size and far less robust appearance, and shorter antennae; they are distinguished from *Caledonotrichia minuta* by having the ventral lobes of gonopods about half as long as dorsal lobes, dorsal lobes less broadly rounded, and sclerotised rods of subgenital process bifid apically, but not dilated as in *Caledonotrichia minor*.

#### Description, male.

Head. Rounded in dorsal view, as in *Caledonotrichia capensis* sp. n. (Fig. 1). Antennae (Fig. 16) with 22–23 flagellomeres; most flagellomeres elongate-cylindrical with ends angled obliquely, not more than 2.5× width. Maxillary palps (Fig. 14) with basal segment short and rounded, segments 2, 3 and 5 cylindrical, segments 3 and 5 length about 3× maximum width, segment 4 subquadrate.

Wings. Forewing length, 1.8–2.0 mm (n=5); forewing without scales.

Genitalia (Figs 27–29, 44). Abdominal segment IX truncate proximally, distal margin of sternite shallowly excavated medially, bearing robust elongate setae. Tergite X, tapered slightly, apical margin concave. Gonopods in ventral view with ventral lobes scarcely longer than wide, dorsal lobes about with length about 2× width, twice length of ventral lobes, mesal process digitiform, well developed, without setae; axillary seta slender, acute apically. Sclerotised rods of subgenital process narrowly bifid apically (arrow in Fig. 27). Phallic apparatus elongate, slender in proximal 2/3, stouter distally, with a slender spiny paramere.

#### Material examined.

Holotype male: New Caledonia: Province Sud, Sarraméa, 220 m, forest stream, loc 10 21°37.883'S, 165°51.958'E, Malaise trap, 18–21.xi.2001, Johanson, Pape & Viklund (MNHP).

Paratypes: New Caledonia: 1 male, collected with holotype; 3 males, Province Sud, W slope Mt. Ningua, Kwé Néco, Stream, at Camp Jacob, 3.7 km WNW summit of Mt. Ningua, on Boulouparis—Thio Road, about 50 m upstream road, 21°43.613'S, 166°06.567'E, 150 m, 29.xi–12.xii.2003, Malaise trap, loc#054, K. A. Johanson (NHRS); 2 males, Province Nord, Wan Pwé On Stream, draining NNE side of Mt. Panié, 3.9 km NW Cascade de Tao, 20°31.820'S, 164°47.016'E, 18.xii.2003, light trap, loc#085, K. A. Johanson (NHRS).

#### Etymology.

In reference to the bilobed apices of the sclerotised rods in the male genitalia.

### Caledonotrichia ouinnica sp. n.

urn:lsid:zoobank.org:act:DC29045A-A990-4F62-BB01-1C4BDD6136B4

http://species-id.net/wiki/Caledonotrichia_ouinnica

Figs 6 15 36–37 45

#### Diagnosis.

Males are recognised by the presence of a tiny jet black spot on the forewing between veins R and M, formed by a cluster of androconial scales; but it is particularly distinguished from all other species by maxillary palps with dense brush of elongate setae on the first segment and bristle-like setae on other segments which give the palps a bottlebrush-like appearance (Fig. 15), including the otherwise closely similar *Caledonotrichia minor* which has maxillary palps of the usual form with fewer and shorter straight setae, the forewings are less attenuate apically and the area of scales over the fork in M larger.

#### Description, male.

Head rounded in dorsal view, as in *Caledonotrichia capensis* sp. n. (Fig. 1). Forewing (Fig. 6) length, 2.2–2.4 mm (n=5); wing acute apically; small cluster of slender jet black androconia proximally. Maxillary palps densely hairy (Fig. 15), with basal 2 segments short and rounded, segment 2 bearing a tuft of long setae, segment 3–5 cylindrical, segment 3 length almost 3× maximum width, segment 4 length about 2× width, segment 5 length approximately 6× width. Antennae with 23–24 flagellomeres, median flagellomeres slender with length around 4–6× width.

Genitalia (Figs 36, 37, 45). Abdominal segment IX rounded anteriorly, sternite slightly cleft medially on posterior margin, a row of elongate setae on distal margin (arrow in Fig. 36). Tergite X tapered to broadly rounded apex. Each gonopod with ventral lobe club-shaped, dense short setae towards tip, dorsal lobe rectangular. Sclerotised rods of subgenital process in ventral view simple, rounded apically. Phallic apparatus elongate, almost length of abdominal segments VII–IX, without associated parameres.

#### Material examined.

Holotype, male: New Caledonia: Province Sud, Mt. Dzumac, source stream of Ouinne River, near crosspoint to mountain track, 22°02.073'S, 166°28.460'E, 810 m, 18.xi–4.xii.2003, Malaise trap, loc#030, K. A. Johanson (MNHP).

#### Paratypes.

New Caledonia. 3 males, collected with holotype, (NHRS); 1 male (dissected) Province Sud, Monts Kwa Ne Mwa, on road between Noumea and Yaté, 1.5 km E Pic Mouirange, 22°12.545'S, 166°40.246'E, 143 m, 9.xi.2003, light trap, loc#018, K. A. Johanson (NHRS); 3 males, 5 females, Province Sud, Mt. Dzumac, source stream of Ouinne River, near crosspoint to mountain track, 22°02.439'S, 166°28.646'E, 805 m, 18.xi–4.xii.2003, Malaise trap, loc#029, K. A. Johanson (NHRS).

#### Etymology.

In reference to the river at the type locality.

### Caledonotrichia minor

Sykora

http://species-id.net/wiki/Caledonotrichia_minor

Figs 5 38–40 46–47

Caledonotrichia minor Sykora (1967: 585–595); Wells (1995: 230).

#### Revised diagnosis.

Males of *Caledonotrichia minor* share with those of *Caledonotrichia charadra*, *Caledonotrichia capensis* sp. n. and *Caledonotrichia ouinnica* sp. n. the presence of androconia on the forewing only (Fig. 5), and are distinguished from these species by the size of the single patch of scales, which forms a small dark area proximally, though larger than the tiny black spot of *Caledonotrichia ouinnica*, whereas *Caledonotrichia charadra* and *Caledonotrichia capensis* sp. n. have large areas; in the male genitalia (Figs 38–40, 46–47), as in *Caledonotrichia charadra* and *Caledonotrichia capensis*, the ventral lobes of the gonopods in ventral view are triangular, but the sclerotised rods of the ventral processes are bilobed and broadly flared apically (arrow in Fig. 39), whereas the other two species have just a small subapical irregularity. The forewing of *Caledonotrichia minor* is not as slender as that of *Caledonotrichia ouinnica*, which tapers to an acute apex.

#### Revised description, male.

Male head rounded as in *Caledonotrichia capensis* sp. n. (Fig. 1).

Antennae with 24–28 flagellomeres (n=5); flagellomeres elongate cylindrical. Maxillary palps with basal 2 segments short and rounded, rest cylindrical: segment 3 about 3× maximum width, segment 4 length about 2× width, and 5 elongate slender, length almost 6× width. Forewing (Fig. 5) length, 1.6–2.1 mm (n=5); small patch of slender scales medially at about one third length, some upright bristles on veins, costal margin hairs straight.

#### Material examined.

Holotype, male: New Caledonia: River near Col d’Amieu (BPBM) (examined).

#### Other material examined:

New Caledonia: 1 male, Nékliai River, 5 km above Mission Station [near Poya], 10.viii.1965, F. Starmühlner (ROM); 1 pharate male pupa, larvae, Nerihouen River, St Ives, Reg. Ponérihouen, 27.viii. 1965, F. Starmühlner (ROM); 30 males, 7 females, Ouenghi River nr Boulouparis, 14.xii.1983, A. Wells (ANIC); 13 males, 8 females, same locality, 19.xii.1983, A. Wells (ANIC); 34 males, 7 females, Bopope, 18.xii.1983, A. Wells (ANIC); 28 males, ~10 km NW Hienghène, small stream, 25.xii.1998, A. Wells (ANIC); 2 males (on slides), Prov. Sud, Sarraméa, 220 m, forest stream, loc 10 21°37.883'S 165°51.958'E, Malaise trap, 18–21.xi.2001, Johanson, Pape & Viklund (NHRS).

#### Remarks.

The mature larva illustrated by Wells (1995: 226, fig. 2) is typically stactobiine, its case a round dome that is attached to the rock surface.

### Caledonotrichia vexilla sp. n.

urn:lsid:zoobank.org:act:D873D57C-42C8-4BBC-A1A4-E8F10DBCDCEB

http://species-id.net/wiki/Caledonotrichia_vexilla

Figs. 2, 7 41–43 48–50

#### Diagnosis.

Males lack scales on wings but otherwise in features of male genitalia resemble closely those of *Caledonotrichia charadra* and *Caledonotrichia capensis* sp. n., having ventral lobes of gonopods triangular but in *Caledonotrichia charadra* dorsal lobes are somewhat rounded, in *Caledonotrichia capensis* they are rectangular, while in *Caledonotrichia vexilla* they are tapered apically; *Caledonotrichia capensis* has the phallic apparatus short with a sharply angled paramere, while *Caledonotrichia vexilla* has the phallic apparatus elongate, about length of 3 abdominal segments, and the paramere spine strongly sinuous.

#### Description, male.

Head (Fig. 2) rectangular in dorsal view, length about one-third the width. Antennae with 24–25 flagellomeres; flagellomeres elongate-cylindrical, with length 2.5–3× width. Maxillary palps with basal 2 segments round, segments 3–5 cylindrical, segment 3 relatively stout, slightly longer than segment 5, segment 5 length about 6× width, apical margin truncate.

Forewing (Fig. 7), length 2.2 mm (n=3); wings without scales, forewing densely hairy, with upright bristle-like hairs on veins, and on distal two-thirds costal margin long slender hairs with strongly curved tips.

Genitalia (Figs 41–43, 48–50). Abdominal segment IX in ventral view shield-shaped, distal margin shallowly excavated medially. Tergite X, subquadrate, apical margin concave. Gonopods bilobed, ventral lobe sharply triangular with row of stout setae on apical margin, dorsal lobe subrectangular, tapered slightly towards outer apical angle, mesal process spur-shaped, sclerotised; axillary seta short. Sclerotised rods of subgenital process with a slight irregularity laterally below apex. Phallic apparatus elongate, sinuous, with a stout, sinuous spiny paramere.

#### Material examined.

Holotype male (on slide): New Caledonia: Parc de Rivière Bleu, approx 1 km W Kaori Giant, 19.xii.1998, A. Wells (MNHP).

Paratypes: New Caledonia. 2 males (1 on slide), collected with holotype (ANIC); 6 males, Parc de Rivière Bleu, Rivière Bleue, approx 1 km W Kaori Giant, 19.xii.1998, A. Wells (ANIC); 4 males, Rivière du Cap, approx 8 km NW Naindai, Bourail to Poya road, 22.xii.1998, A. Wells (ANIC).

#### Other material examined:

New Caledonia 1 pharate male pupa (damaged), 3 cases, Rivière Bleue, bridge forest road, 21.vii.1965, F. Starmühlner (ROM).

#### Etymology.

In reference to the vexatious problem of distinguishing this species from others primarily on the basis of internal features.

#### Remarks.

Males of this small jet black species were collected in bright sunlight as they ran about on an emergent rock in the stream. The three cases collected with the single pharate male pupa from Rivière Bleue, resemble closely those described by Wells (1985: 11) for *Caledonotrichia illiesi*, having a broad flat margin to the case; however, they lack dorsal vents on the dome seen in that species.

### Caledonotrichia capensis sp. n.

urn:lsid:zoobank.org:act:321477EA-EB4F-457F-BF45-0963BA37E082

http://species-id.net/wiki/Caledonotrichia_capensis

Figs 1, 8 51–53 64–65

#### Diagnosis.

Males of this species are readily recognised by the band of elongate-ovoid black scales that stretches almost the length of the forewing; in features of genitalia they closely resemble *Caledonotrichia minor* and *Caledonotrichia vexilla* sp. n. Females not associated.

#### Description, male.

Head rounded (Fig. 1). Antennae with 24 flagellomeres; flagellomeres elongate cylindrical, longest with length about 3× width. Maxillary palps with basal 2 segments short and rounded, rest cylindrical: segment 3 about 4× maximum width, segment 4 length about 2.5× width, segment 5 elongate slender, length almost 6× width.

Wings. Forewing (Fig. 8) length, 1.0–1.7 mm (n=5); bearing elongate patch of black scales (androconia) medially reaching almost from leading proximal angle to about two-thirds wing length, and separate small patch close to proximal margin; costal margin hairs straight. Hind wing bearing small rather scattered black scales, more slender than those of forewing.

Genitalia (Figs 51–53, 64–65). Abdominal segment IX rounded proximally, apical margin cleft medially. Tergite X with lateral margins slightly rounded, apically concave. Gonopods with ventral lobes triangular, apical margin slightly concave, dorsal lobes elongate rectangular; axillary seta not apparent. Sclerotised rods of subgenital process in ventral view with small cap-like irregularity apically and another subapically, in lateral view dilated apically. Phallic apparatus elongate, slender, strongly curved at about two-thirds length, with stout parameres.

#### Material examined.

Holotype male: New Caledonia: Rivière du Cap, Pont du Cap, ~8 km NW Naindai on Bourail-Poya road, 22.xii.1998, A. Wells (MNHP).

Paratypes: 5 males, same data as for holotype (one on slide) (ANIC).

#### Etymology.

Named for the type locality, Rivière du Cap.

#### Remarks.

The specimens of this species were collected as they ran about in sunlight on the surfaces of emergent rocks in the stream.

### Caledonotrichia charadra

Kelley

http://species-id.net/wiki/Caledonotrichia_charadra

Figs 9–10 54–56

Caledonotrichia charadra Kelley (1989: 194).

#### Revised diagnosis.

Like *Caledonotrichia capensis* males have scale patches on both fore- and hind wings (Figs 9–10), a feature that distinguishes it from *Caledonotrichia minor* and *Caledonotrichia ouinnica*, both of which have scale patches on the forewings only; in the genitalia (Figs 54–56) the triangular shape of ventral lobes of the gonopods of *Caledonotrichia charadra* closely resembles the arrangement in *Caledonotrichia capensis* sp. n. and *Caledonotrichia vexilla* sp. n. but in *Caledonotrichia charadra* the dorsal lobes are quadrilateral, rather than elongate rectangular or subrectangular as in those two species, and the paramere associated with the phallic apparatus is more gently curved.

#### Additional information, male.

Male head rounded as in *Caledonotrichia capensis*. Male antennae with 24–26 flagellomeres; flagellomeres elongate rectangular. Male maxillary palps as for *Caledonotrichia capensis*. Male forewing length, 1.5–1.9 mm (n=10). Fore- and hind wings both bear rectangular patch of black scales proximally (Figs 9–10), although these may be shed or lost due to abrasion.

#### Remarks.

Several features noted on examination of the type specimen of *Caledonotrichia charadra* that were not mentioned by Kelley (1989) were scales on wings and the subapical irregularity on the ventral processes, that gives the apex a hooked appearance when seen in lateral view. In addition, while the type specimen has scales on the forewing only, we are assuming that the scales were lost from the hind wing as, in other respects, the more recently collected specimens conform to the type but have scales on both wings.

#### Material examined.

Holotype male: New Caledonia: mountain stream up Boulari River (BPBM) [entire animal macerated and stored in vial].

#### Other material examined.

1 male, Ouenghi River nr Boulouparis, 14.xii.1983, A. Wells (ANIC); 37 males, 1 female, Parc de la Rivière Bleue, approx 1 km W Kaori Giant, 19.xii.1998, A. Wells (ANIC).

### Caledonotrichia extensa

Kelley

http://species-id.net/wiki/Caledonotrichia_extensa

Figs 3 11–12, 19 57–58 66, 70

Caledonotrichia extensa Kelley (1989: 195).

#### Revised diagnosis.

As noted by Kelley (1989) *Caledonotrichia extensa* is distinctive in being larger than any other species; as in males of *Caledonotrichia nyurga* antennae are extremely long, just exceeding length of body, but in contrast to that species the segments (Fig. 19) tend to be more elongate, inverted urn-shaped, not bead-like, and the longest segments are about 6× as long as wide; maxillary palps (Figs 11–12) with segments 1 and 2 round, segments 4 and 5 length about 8× width, segment 3 length about 1.5× length of each of terminal segments, swollen apically and bearing area of sensilla apico-dorsally (Fig. 12); male genitalia are clearly visible under a dissecting microscope, with abdominal sternite IX deeply concave distally, ventral lobes of gonopods rounded, bearing row of stout dark setae on mesal margin, not narrowly membranous and leaf-like as in *Caledonotrichia nyurga*, or slender and finger-like as in *Caledonotrichia sykorai* sp. n.

#### Additional information, male.

Head subquadrate; antennae (Fig. 19) with 25–26 flagellomeres (n=10); scape elongate-rounded, pedicel short, rounded, other flagellomeres elongate-cylindrical to flask-shaped, bearing numerous *sensilla coeloconica*.

Forewing (Fig. 3) without scales or specialised setae, venation more complete than in other congeners; length: 2.2–3.6 mm (n=10).

Genitalia (Figs 57–58, 66). Abdominal segment IX in ventral view almost triangular, strongly tapered and narrowly rounded anteriorly, a deep concavity on posterior margin of sternite. Tergite X narrow, elongate, straight sided, rounded apically. Gonopods with ventral lobe length 1.5X width, bearing stout setae on mesal margin and apically, dorsal lobe elongate club-shaped, a row of sclerotised short setae subapico-mesally, most distal pair bent sharply, axillary seta long, slender, apically acute. Sclerotised rods of subgenital process straight, slightly sculptured apically. Phallic apparatus straight, elongate, slender, with a dorsal crease along two-thirds length and with associated spine-covered apical membrane.

Case of mature larva (Fig. 70). Cigar-shaped, with a pair of dorsal vents and at each end a bract-like overhang. Cased larvae of *Caledonotrichia extensa* were associated via pharate adults, and demonstrate that unlike the larvae of *Caledonotrichia illiesi* that live in fixed dome-shaped cases, larvae of *Caledonotrichia extensa* are mobile, carrying their cases about. These portable cases are cigar-shaped, tapered at each end and comprise two identical secretion valves, clearly with an upper and lower side. In common with cases of *Caledonotrichia illiesi*, however, cases of *Caledonotrichia extensa* have dorsal vents, although in *Caledonotrichia extensa* these are larger openings, situated near the ends of the dorsal seam of the two valves and opening away from the case.

#### Material examined.

Holotype male: New Caledonia: mountain stream up Boulari River (BPBM).

#### Additional material examined.

New Caledonia: 1 cased larva, Ouarou River, source of Tchamba R., N of Ponerinouen, 25.viii.1965, F. Starmühlner (ROM); prepupae, pupae, middle Tchamba R., below Tchamba, 26.viii.1965, F. Starmühlner (ROM); 8 males, Nerihouen River, St Ives, Reg. Ponérihouen, 27.viii. 1965, F. Starmühlner (ROM); larvae, pupae, St Ives Reg. Ponérihouen, 27.viii. 1965, F. Starmühlner (ROM); larvae, pupae, 1 female, 3 km from mouth of Mou River, Reg. Ponérihouen, 28.viii. 1965, F. Starmühlner (ROM); 1 prepupa, river Col d’Boa, [no date], F. Starmühlner (ROM); 1 male, Yaté turnoff, 24.viii.1973, A.G. McFarlane & R.A. Savill; 1 male, 1 female, 4 km SW Col de Mouirange, 20 m, 10.viii.1979, G.M. Nishida (BPBM); larvae, pupae, Boghen, Oct. 1996, N. Mary (ANIC); 45 males, Rivière du Cap, Pont du Cap, approx 8 km NW Naindai on Bourail-Poya road, 22.xii.1998, A. Wells; 3 males, stream, approx 15 km SW Thio on Boulouparis-Thio road, 28.xii.1998, A. Wells (ANIC); pupae, River Ni, 25 July 2000, N. Mary (ANIC); 1 male, Province Nord, Ponandou Tiôgé River at Kögi, 3.9 km SSW Touho, 20°49.043'S, 165°13.551'E, 25 m, 26.xii.2003, light trap, loc#100, K. A. Johanson (NHRS).

### Caledonotrichia nyurga

Oláh & Johanson

http://species-id.net/wiki/Caledonotrichia_nyurga

Figs 18 61–63 69

Caledonotrichia nyurga Oláh & Johanson (2010: 101).

#### Revised diagnosis.

Males of this species are recognised in mixed collections by the clearly visible genitalia (Figs 61–63, 69), a consequence of the deep excision of the posterior margin of sternite IX, and also by the moniliform distal segments of the antennae. *Caledonotrichia nyurga* is smaller-bodied than both *Caledonotrichia extensa* and the otherwise similar *Caledonotrichia sykorai* sp. n. with which it shares the feature of a pronounced mesal process on the posterior margin of sternite VII, though in the latter species the mesal process is far shorter than in *Caledonotrichia nyurga* and elongate triangular. *Caledonotrichia nyurga* also differs from *Caledonotrichia sykorai* by the very slender anterior extension of abdominal segment IX, broader ventral lobes of gonopods, and the less sharply excised abdominal tergite IX.

Additional information, male. Head, rounded as in *Caledonotrichia capensis*. Antennae (Fig. 18) with 25–39 flagellomeres; flagellomere shape variable; distal flagellomeres moniliform, proximal flagellomeres elongate cylindrical. Maxillary palps with basal 2 segments short and rounded, rest cylindrical: segment 3 slightly shorter than segment 4, segment 5 slender, elongate with length about 10× as long as width and equal to length of segments 3 and 4 together. Forewing length, 1.4–2.0 mm (n=10), apically less sharply tapered than other congeners.

#### Material examined.

New Caledonia: Holotype male, Province Sud, W slope Mt. Ningua, Kwé Néco, Stream, at Camp Jacob, 3.7 km WNW summit of Mt. Ningua, on Boulouparis–Thio Road, about 50 m upstream road, 21°43.613'S, 166°06.567'E, 150 m, 29.xi–12.xii.2003, Malaise trap, loc#054, K. A. Johanson (MNHN); 3 males (on slides), Province Sud, Rivière Bleue, 282 m, stony river, loc 4, 22°05.705'S, 166°38.225'E, Malaise trap, 13–16.xi.2001, Johanson, Pape & Viklund (NHRS); 2 males (1 male on slide), Province Sud, Sarraméa, 2907 m, stony forest stream, loc 13 21°37.097'S 165°49.351'E, Malaise trap, 18–21.xi.2001, Johanson, Pape & Viklund (NHRS); 4 males, Province Nord, Mt. Panié, 350 m, loc. 16 rocky river downstream waterfall, 20°35.864'S, 164°49.780'E, Malaise trap, 22–26.xi.2001, Johanson, Pape & Viklund (NHRS); 4 males, Province Sud, Monts Kwa Ne Mwa, on road between Noumea and Yaté, Rivière des Pirogues, 22°11.225'S, 166°43.338'E, 100 m, 7.xi.2003, light trap, loc#016, K. A. Johanson (NHRS); 1 male, Province Sud, Mt. Dzumac, source stream of Ouinne River, near crosspoint to mountain track, 22°02.439'S, 166°28.646'E, 805 m, 18.xi–4.xii.2003, Malaise trap, loc#029, K. A. Johanson (NHRS); 10 males, 11 females, Province Sud, Mt. Dzumac, source stream of Ouinne River, near crosspoint to mountain track, 22°02.073'S, 166°28.460'E, 810 m, 18.xi–4.xii.2003, Malaise trap, loc#030, K. A. Johanson (NHRS); 4 males, Province Sud, Mt. Dzumac, source stream of Ouinne River, downstream crosspoint to mountain track, 22°01.997'S, 166°28.486'E, 795 m, over about 30 m waterfall, 18.xi–4.xii.2003, Malaise trap, loc#031, K. A. Johanson (NHRS); 15 males, Province Sud, W slope Mt. Ningua, Kwé Néco, Stream, at Camp Jacob, 3.7 km WNW summit of Mt. Ningua, on Boulouparis–Thio Road, about 50 m upstream road, 21°43.613'S, 166°06.567'E, 150 m, 29.xi–12.xii.2003, Malaise trap, loc#054, K. A. Johanson (NHRS); 1 male, Province Sud, Creek Froid, 10 m upstream bridge on La Foa–Koindé road, 200 m W crossroad to Ouipouin, 21°38.581'S, 165°56.672'E, 180 m, 4.i.2004, light trap, loc#105, K. A. Johanson (NHRS).

#### Holotype male (examined):

New Caledonia, Provence Sud, W. slope of Mt Ningua, Kwe Néco Stream, at Camp Jacob, 3.7 km WNW summit of Mt. Ningua, on Boulouparis-Thio Road, 21°43.613'S, 166°06.567'E, 29.xi–12.xii.2003, K. A. Johanson (alcohol, MNHP).

### Caledonotrichia sykorai sp. n.

urn:lsid:zoobank.org:act:7D4B7BA3-BCDC-4C2B-9245-267182788C4C

http://species-id.net/wiki/Caledonotrichia_sykorai

Figs 17 59–60 67–68 73

#### Diagnosis.

Closely resembling *Caledonotrichia nyurga*, but males differing in having the posterior margin of sternite IX only shallowly concave, the anterior extension of abdominal segment IX shorter and broader; and gonopods with ventral lobes narrower and dorsal lobes stout, as wide at apex as close to base; both *Caledonotrichia sykorai* and *Caledonotrichia nyurga* can be separated from *Caledonotrichia extensa* by their smaller size and presence of the elongate ventral process on the posterior margin of sternite VII. Females have the apical margin of abdominal sternite VII rounded, without the membranous collar seen in *Caledonotrichia illiesi* and *Caledonotrichia minuta*.

#### Description, male, female.

Head rounded, as in *Caledonotrichia capensis* (Fig. 1). Antennae (Fig. 17) with 24–25 flagellomeres, of form seen in *Caledonotrichia illiesi* and most other species of *Caledonotrichia* with flagellomeres elongate cylindrical. Maxillary palps similar to those in males of *Caledonotrichia nyurga*, but terminal segment shorter, relatively, but still exceeding length of segments 3 or 4 which are subequal.

Wings. Forewing length, 2.2–2.8 mm (n=5).

Male genitalia (Figs 59, 60, 67, 68). Posterior margin of abdominal sternite VII bearing elongate triangular, mesal process. Abdominal segment IX ventrally subquadrate posteriorly, anteriorly rounded triangular; dorsally with deep parallel-sided excision. Tergite X convex apically. Sclerotised rods of ventral processes stout, simple. Gonopods with ventral lobe in form of narrow filaments, dorsal lobes stout, without mesal processes. Ventral processes stout, strongly curved at base. Phallic apparatus with associated slender parameres.

Female terminalia (Fig. 73). Forming a slender, telescopic oviscapt, but lacking the medial process on sternite VII seen in *Caledonotrichia illiesi* and *Caledonotrichia nyurga*.

#### Material examined.

Holotype male: New Caledonia: Province Sud, stream crossing way to sanatorium 2.3 km E St Laurent, ca. 150 m upstream bridge, 22°04.484'S, 166°19.910'E, loc 027, Malaise trap, 17–19.x.2006, K. A. Johanson & M. Espeland (NHRS).

**Paratypes:** New Caledonia: 6 males, 30 females, Province Sud, Col d’Amieu, 319 m, small stony river, loc 23, 21°34.720'S, 165°49.620'E, Malaise trap, 30.xi–5.xii.2001, Johanson, Pape & Viklund (NHRS); 2 males (on slides), Province Sud, Monts des Koghis, ca 800 m S Koghi Restaurant, 22.18406°S, 166.50383°E, 420 m, 11–26.xi.2003, Malaise trap, loc#022, K. A. Johanson (NHRS); 1 male, 1 female, Province Sud, Hwa Hace Mt., Hwa Motu River, at Pont Wamuttu, 1.0 km E Nassirah, about 200 m upstream bridge, 21°48.094'S, 166°04.298'E, 137 m, 20.xi–12.xii.2003, Malaise trap, loc#034, K. A. Johanson (NHRS); 4 males, 4 females (2 males 1 female on slide), Province Sud, stream crossing way to sanatorium 2.3 km E St Laurent, ca. 150 m upstream bridge, 22°04.484'S, 166°19.910'E, loc 027, Malaise trap, 17–19.x.2006, K. A. Johanson & M. Espeland (NHRS).

#### Etymology.

*Sykorai*, named for Dr Jan Sykora, who described the first two species in this genus**.**

##### Key to adult males of *Caledonotrichia*

**Table:** 

1	Forewing bearing at least one area of scales (scent scales or androconia) (Figs 5, 6, 8, 9)	2
–	Forewing without scales (Figs 3, 4, 7)	5
2	Scale patches on forewing large (Figs 8, 9); ventral lobe of gonopods more or less triangular in ventral view with apical margin slightly concave (Figs 51, 54); ventral processes in ventral view with subapical irregularity (Figs 51, 54)	3
–	Scale patches on forewing small (Figs 5, 6); ventral lobe of gonopods with apical margin rounded (Figs 36, 39); ventral processes with apices rounded, or bifid and dilated (Figs 36, 39, 46)	4
3	Scales on forewing in long band extending in a band for about two-thirds the length of wing, and small patch anteriorly of jugal region (Fig. 8); dorsal lobe of gonopods rectangular in dorsal view (Figs 53, 65)	*Caledonotrichia capensis*, sp. n.
–	Scale patches on only proximal one-third of forewing (Fig. 9); dorsal lobe of gonopods quadrangular in dorsal and ventral views (Figs 54, 56)	*Caledonotrichia charadra* Kelley, 1989
4	Patch of scales on forewing tiny, appearing as small, jet black spot (Fig. 6); maxillary palps bearing dense cover of curved setae, giving bottle brush-like appearance (Fig. 15); ventral lobe of gonopods round in ventral view (Fig. 36)	*Caledonotrichia ouinnica*, sp. n.
–	Patch of scales on forewing more diffuse, not forming a small jet black spot (Fig. 5); maxillary palps bearing sparse, short straight setae; ventral lobe of gonopods in ventral view more or less triangular, with apical margin rounded (Figs 39, 46)	*Caledonotrichia minor* Sykora, 1967
5	Abdominal segment IX in ventral view anteriorly triangular (Figs 57, 60, 61); abdominal sternite VII usually bearing slender, elongate caudally directed process on proximal margin (Figs 60, 61, 63)	6
–	Abdominal segment IX shield-shaped, or anteriorly subquadrate (e.g. Figs 21, 24, 27); abdominal sternite VII without ventral process (Fig. 49)	8
6	Abdominal sternite VII without digitate process on proximal margin; ventral lobe of gonopods well developed, club-shaped, bearing row of short, stout, dark setae (Fig. 57)	*Caledonotrichia extensa* Kelley, 1989
–	Abdominal sternite VII bearing digitate process on proximal margin (Figs 60, 61, 63); ventral lobe of gonopods slender, leaf-like to narrow (Figs 60, 61, 67, 69)	7
7	In ventral and lateral view abdominal segment IX greatly extended anteriorly in a slender, elongate process (Figs 61, 63, 69); antennae elongate, with 37 flagellomeres, distal segments no longer than wide, urn-shaped (Fig. 18)	*Caledonotrichia nyurga* Oláh & Johanson, 2010
–	In ventral view abdominal segment IX slightly extended anteriorly, coarsely triangular (Figs 60, 67); antennae with 24 flagellomeres with length 1.5–2.5× width (Fig. 17)	*Caledonotrichia sykorai*, sp. n.
8	Antennae with 33–38 flagellomeres; ventral lobes of gonopods rounded, only slightly longer than wide (Figs 21, 30); seta dorsally on ventral lobe of gonopods elongate, slender, a round knob at tip (Figs 21, 22)	*Caledonotrichia illiesi* Sykora, 1967
–	Antennae with 22–25 flagellomeres; ventral lobes of gonopods rounded (Figs 24, 27, 33, 44) or triangular (Figs 41, 48); if present seta dorsally on ventral lobe of gonopods elongate, slender, without knob at tip (Figs 24, 27)	9
9	Ventral lobe of gonopods triangular, setae on posterior margin stout (Figs 41, 48); dorsal lobe tapered apically (Figs 43, 49, 50)	*Caledonotrichia vexilla*, sp. n.
–	Ventral lobe of gonopods rounded apically, setae on posterior margin fine, short (Figs 24, 27, 33); dorsal lobe bat-shaped or broadly rounded (Figs 23, 29, 35)	10
10	Ventral lobe of gonopods about half length of dorsal lobe (Fig. 27); dorsal lobe broad, longer than wide (Figs 29, 44), mesal processes close to proximal margin; sclerotised rods of subgenital processes bifid apically (Figs 27, 44), not flared	*Caledonotrichia bifida*, sp. n.
–	Ventral lobe of gonopods about same length as dorsal lobe; dorsal broadly rounded (Figs 23, 35), with mesal process situated in middle of lobe; sclerotised rods of subgenital processes with small lateral irregularity close to apex (Fig. 26)	*Caledonotrichia minuta*, sp. n.

## Supplementary Material

XML Treatment for Caledonotrichia

XML Treatment for Caledonotrichia illiesi

XML Treatment for Caledonotrichia minuta

XML Treatment for Caledonotrichia bifida

XML Treatment for Caledonotrichia ouinnica

XML Treatment for Caledonotrichia minor

XML Treatment for Caledonotrichia vexilla

XML Treatment for Caledonotrichia capensis

XML Treatment for Caledonotrichia charadra

XML Treatment for Caledonotrichia extensa

XML Treatment for Caledonotrichia nyurga

XML Treatment for Caledonotrichia sykorai
